# Suppression of epithelial-mesenchymal transition in hepatocellular carcinoma cells by Krüppel-like factor 4

**DOI:** 10.18632/oncotarget.8831

**Published:** 2016-04-18

**Authors:** Qi Li, Weifeng Song, Weiyu Wang, Shanshan Yao, Chuan Tian, Xun Cai, Liwei Wang

**Affiliations:** ^1^ Department of Oncology, Shanghai General Hospital, Shanghai Jiao Tong University School of Medicine, Shanghai 200080, China; ^2^ Department of Oncology, Shanghai East Hospital, Tongji University, Shanghai 200120, China; ^3^ Department of Interventional Radiology, Affiliated Anhui Provincial Hospital, Anhui Medical University, Hefei 230001, China

**Keywords:** Krüppel-like factor 4, epithelial-mesenchymal transition, hepatocellular carcinoma, microRNA

## Abstract

Hepatocellular carcinoma (HCC) is one of the most malignant and lethal human cancers. Epithelial-mesenchymal transition (EMT) enhances the carcinogenesis of HCC, and therapies targeting EMT appear to be promising treatments. We have previously shown that Krüppel-like Factor 4 (KLF4) suppressed EMT of HCC cells through downregulating EMT-associated proteins. Here, we examined the roles of microRNAs (miRNAs) in KLF4-regulated EMT in HCC cells. KLF4 induced expression of 3 miRNAs (miR-153, miR-506 and miR-200b) that targeted 3′-UTR of Snail1, Slug and ZEB1 mRNAs, respectively, to inhibit protein translation in HCC cells, which was confirmed by promoter luciferase assay. Expression of either miRNA significantly inhibited HCC cell growth and invasiveness, while the effect of combined expression of all 3 miRNAs was more pronounced. Furthermore, overexpression of antisense of all 3 miRNAs abolished the inhibitory effect of KLF4 on HCC cell growth and invasiveness. Together, our data suggest that KLF4 inhibits EMT-enhanced HCC growth and invasion, possibly through reducing EMT-related proteins Snail1, Slug and ZEB1 via increasing miR-153, miR-506 and miR-200b.

## INTRODUCTION

The malignancy of hepatocellular carcinoma (HCC) largely results from its aggressive characteristics [1–6]. Hence, targeting therapies upon the invasiveness and metastases of HCC may be effective treatments.

The zinc finger protein Krüppel-like factor 4 (KLF4) regulates gene transcription and cell fate in a context-dependent manner, and has been shown to promote cell differentiation, and to suppress tumor growth stem cell and malignant progression [7–9]. First of all, KLF4 is dispensable for early development [7–9]. Moreover, when KLF4 is expressed in adult somatic cells with other Yamanaka factors [10, 11], KLF4 has been shown to promote the formation of induced pluripotent stem (IPS) cells [12]. For example, KLF4 is found to suppress cell proliferation through modulation of p21Waf1/Cip1 [13]. A variety of growth-suppressive signals activate KLF4, e.g. cell-contact inhibition, serum starvation, DNA damage, and cell differentiation [7–9]. These *in vitro* results suggest KLF4 may suppress cell proliferation rates in cancer cells, including HCC [14–17]. Nevertheless, the underlying mechanisms are not completely understood. We have previously shown that KLF4 binds to the promoter of Vitamin D receptor (VDR) to regulate its expression [18]. Moreover, the levels of KLF4 are reduced and the levels of VDR are increased in HCC cell lines and primary tumor samples [18]. Furthermore, expression of KLF4 in HCC cells sensitizes them to the anti-proliferative effects of VD3, possibly through regulation of epithelial-mesenchymal transition (EMT)-associated events related to cell metastases and growth [18]. Here, we studied how KLF4 may regulate EMT events in HCC.

The role of microRNAs (miRNAs) in the carcinogenesis have been extensively studied previously, especially their involvement in regulation of EMT-associated proteins, Snail1, Slug, ZEB1 and ZEB2. For example, miR-200 family has been shown to inhibit ZEB1 and ZEB2 [19–23], miR-506 has been shown to block Slug translation [24–27], and miR-153 has been shown to suppress Snail1 and ZEB2 [28]. However, whether these miRNAs may be regulated by KLF4 has not been acknowledged.

Here, we examined the involvement of miRNAs in KLF4-suppressed EMT in HCC cells, and the underlying mechanisms.

## RESULTS

### KLF4 increases levels of miR-153, miR-506 and miR-200b in HCC cells

**Figure 1:**
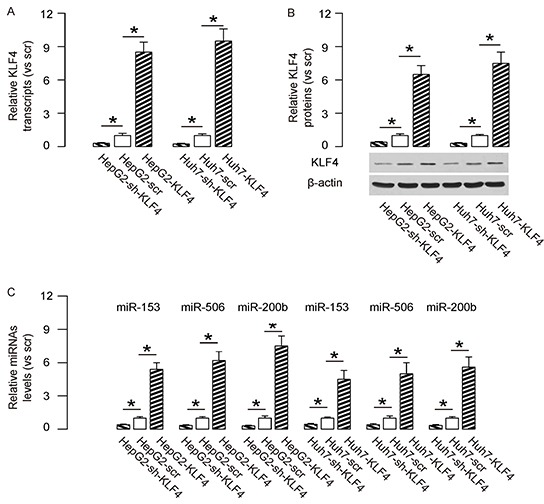
KLF4 increases levels of miR-153, miR-506 and miR-200b in HCC cells We overexpressed KLF4 or depleted KLF4 by shRNAs in two human HCC cell lines, HepG2 and Huh7. **A–B.** KLF4-modifed cells were examined for KLF4 in both HCC lines at the mRNA levels (A), and at protein levels (B). and **C.** The levels of miR-153, miR-506 and miR-200b were shown in KLF4-modifed HCC cell lines. *p<0.05. N=5.

In order to evaluate the effects of KLF4 on the metastases of HCC cells, we overexpressed KLF4 or depleted KLF4 by shRNAs in two human HCC cell lines, HepG2 and Huh7. We found that expression of sh-KLF4 in both HCC lines significantly decreased the mRNA levels (Figure 1A), and protein levels of KLF4 (Figure 1B), while expression of KLF4 in both lines significantly increased the mRNA levels (Figure 1A), and protein levels of KLF4 (Figure 1B). Thus, these KLF4-modified HCC cell lines could be used to exam the KLF4 effects. We have previously reported that KLF4 suppressed the levels of EMT-related proteins, Snail1, Slug and ZEB1, in HCC cells. Here we aimed to figure out whether KLF4 may regulate the expression of these EMT-associated proteins through miRNAs. From all the miRNA candidates, we specifically found that KLF4 overexpression increased the levels of miR-153, miR-506 and miR-200b in both HCC cell lines, while KLF4 depletion decreased the levels of miR-153, miR-506 and miR-200b in both HCC cell lines (Figure 1C). Hence, these miRNAs were analyzed for their associations with EMT-proteins.

### Targeting and inhibition of translation of Snail1 by miR-153, Slug by miR-506 and ZEB1 by miR-200b in HCC cells

**Figure 2:**
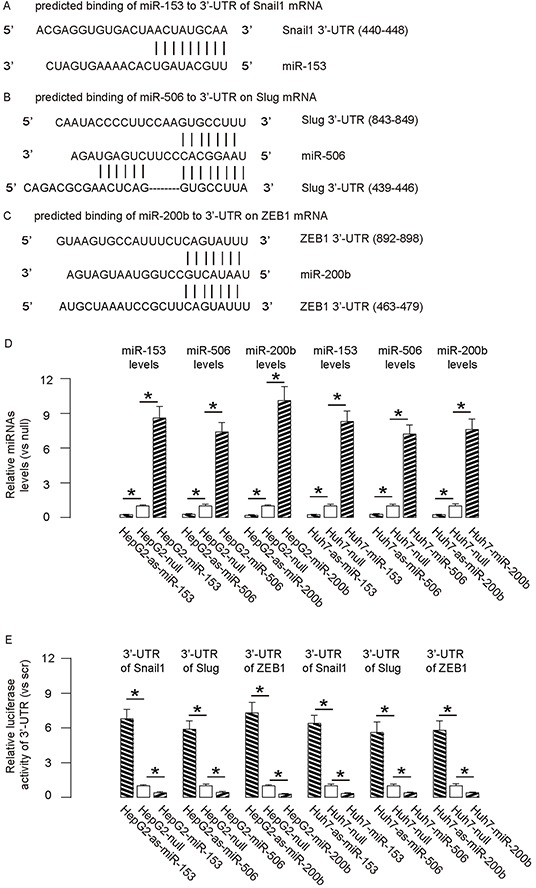
Targeting inhibition of translation of Snail1 by miR-153, Slug by miR-506 and ZEB1 by miR-200b in HCC cells **A–C.** By bioinformatics analyses, we found that miR-153 bound to 3′UTR of Snail1 mRNA at 440-448 base site (A), miR-506 bound to 3′UTR of Slug mRNA at both 439-446 and 843-849 base sites (B), and miR-200b bound to 3′UTR of ZEB1 mRNA at both 463-479 and 892-898 base sites (C) **D.** We either overexpressed miR-153, miR-506 and miR-200b, or inhibited miR-153, miR-506 and miR-200b in both HCC cell lines. These HCC cells were also transfected with a plasmid carrying a null sequence as a control (null). The overexpression or inhibition of these miRNAs in both HCC cell lines was confirmed by RT-qPCR. **E.** The miR-153-modified HCC cells were then transfected with 1μg of Snail1-3′UTR luciferase-reporter plasmid. The miR-506-modified HCC cells were transfected with 1μg of Slug-3′UTR luciferase-reporter plasmid. The miR-200b-modified HCC cells were transfected with 1μg of ZEB1-3′UTR luciferase-reporter plasmid. The luciferase activities were quantified in these cells. *p<0.05. N=5.

By bioinformatics analyses, we found that miR-153 bound to 3′UTR of Snail1 mRNA at 440-448 base site (Figure 2A), miR-506 bound to 3′UTR of Slug mRNA at both 439-446 and 843-849 base sites (Figure 2B), and miR-200b bound to 3′UTR of ZEB1 mRNA at both 463-479 and 892-898 base sites (Figure 2C). In order to confirm that these specific bindings (miR-153/Snail1, miR-506/Slug, miR-200b/ZEB1) are functional, we either overexpressed miR-153, miR-506 and miR-200b, or inhibited miR-153, miR-506 and miR-200b in both HCC cell lines. These HCC cells were also transfected with a plasmid carrying a null sequence as a control. Co-expression of a GFP reporter in these plasmids allow purification of transfected cells by flow cytometry. The overexpression or inhibition of either miRNA in both HCC cell lines was confirmed by RT-qPCR (Figure 2D). The miR-153-modified HCC cells were then transfected with 1μg of Snail1-3′UTR luciferase-reporter plasmid. The luciferase activities were quantified in these cells, suggesting that miR-153 targets 3′UTR of Snail1 mRNA to inhibit its translation (Figure 2E). The miR-506-modified HCC cells were transfected with 1μg of Slug-3′UTR luciferase-reporter plasmid. The luciferase activities were quantified in these cells, suggesting that miR-506 targets 3′UTR of Slug mRNA to inhibit its translation (Figure 2E). Similarly, the miR-200b-modified HCC cells were then transfected with 1μg of ZEB1-3′UTR luciferase-reporter plasmid. The luciferase activities were quantified in these cells, suggesting that miR-200b targets 3′UTR of ZEB1 mRNA to inhibit its translation (Figure 2E).

### MiR-153, miR-506 and miR-200b overexpression inhibits HCC cell growth

**Figure 3:**
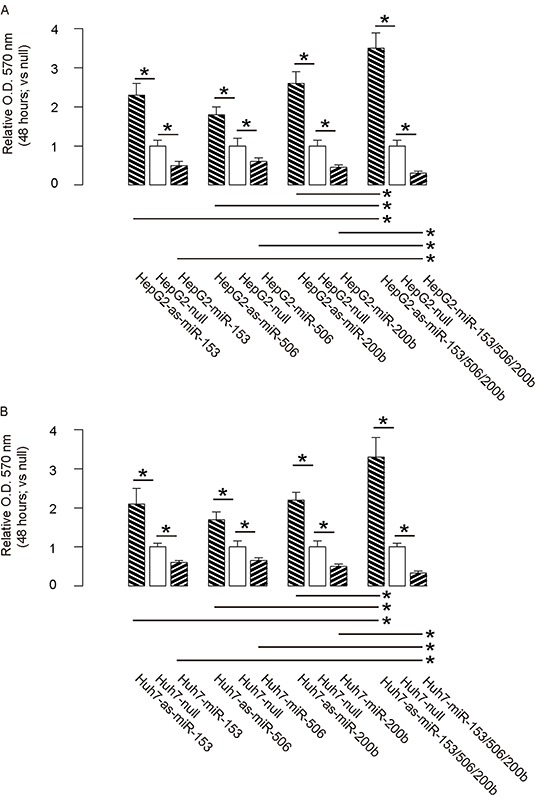
MiR-153, miR-506 and miR-200b overexpression inhibits HCC cell growth **A–B.** The effects of modifications of miR-153, miR-506 and miR-200b on cell growth in an MTT assay, in both HepG2 cells (A) and Huh7 cells (B) *p<0.05. N=5.

Then we examined the effects of miR-153, miR-506 and miR-200b on HCC cell growth in an MTT assay. We found that overexpression of either miRNA in HCC cells significantly decreased cell growth, while depletion of either miRNA in HCC cells significantly increased cell growth, in both HepG2 cells (Figure 3A) and Huh7 cells (Figure 3B). Moreover, the effects of expression of all 3 miRNAs or all 3 as-miRNAs in HCC cells were significantly pronounced, compared to the effects of expression of either single miRNA or as-miRNA, respectively (Figure 3A–3B). These data suggest that miR-153, miR-506 and miR-200b overexpression inhibits HCC cell growth.

### MiR-153, miR-506 and miR-200b overexpression suppresses HCC cell invasion

**Figure 4:**
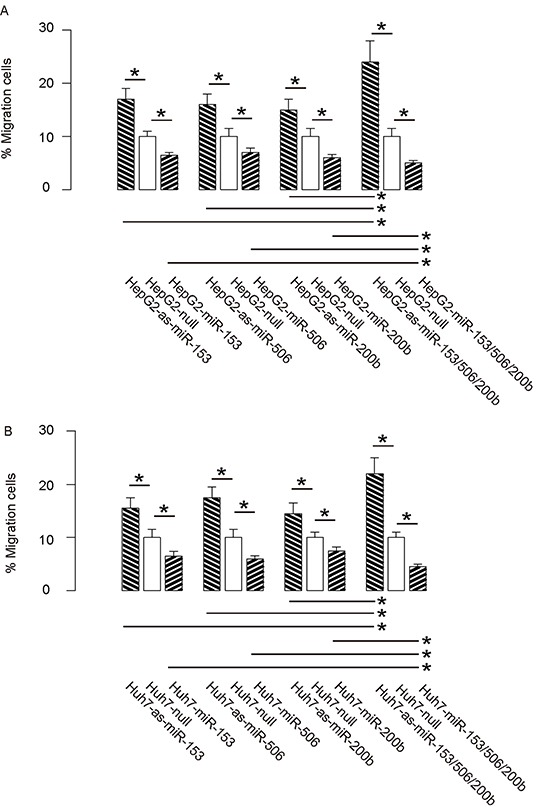
MiR-153, miR-506 and miR-200b overexpression suppresses HCC cell invasion **A–B.** The effects of modifications of miR-153, miR-506 and miR-200b on cell invasiveness in a transwell cell migration assay, shown by the percentage of migrated cells in total cells, in either HepG2 cells (A), or Huh7 cells (B) *p<0.05. N=5.

Then we examined the effects of miR-153, miR-506 and miR-200b on HCC cell invasiveness in a transwell cell migration assay. We found that overexpression of either miRNA in HCC cells significantly decreased cell invasion, while depletion of either miRNA in HCC cells significantly increased cell invasion, in both HepG2 cells (Figure 4A) and Huh7 cells (Figure 4B). Moreover, the effects of expression of all 3 miRNAs or all 3 as-miRNAs in HCC cells were significantly pronounced, compared to the effects of expression of either single miRNA or as-miRNA, respectively (Figure 4A–4B). These data suggest that miR-153, miR-506 and miR-200b overexpression suppresses HCC cell invasion.

### MiR-153, miR-506 and miR-200b depletion abolishes the suppressive effects of KLF4 on HCC cell growth and invasion

**Figure 5:**
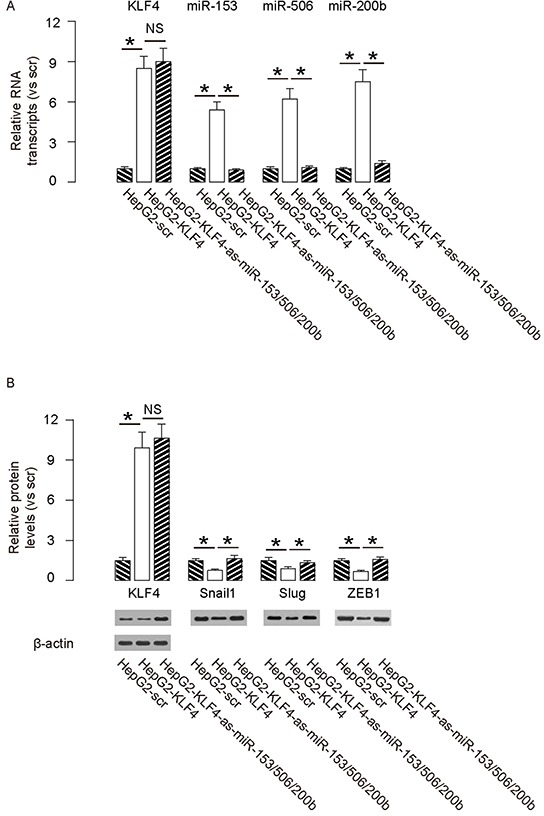
Preparation of miR-153, miR-506 and miR-200b overexpressing HepG2 cells In order to confirm that KLF4 may affect the HCC cell growth and invasion through miR-153, miR-506 and miR-200b, we overexpressed the antisense for miR-153, miR-506 and miR-200b in KLF4-expressing HepG2 cells, and compared to HepG2-KLF4 cells and HepG2-scr cells. **A–B.** The levels of mRNA (A) and protein (B) of KLF4, Snail1, Slug and ZEB1. *p<0.05. NS: non-significant. N=5.

**Figure 6:**
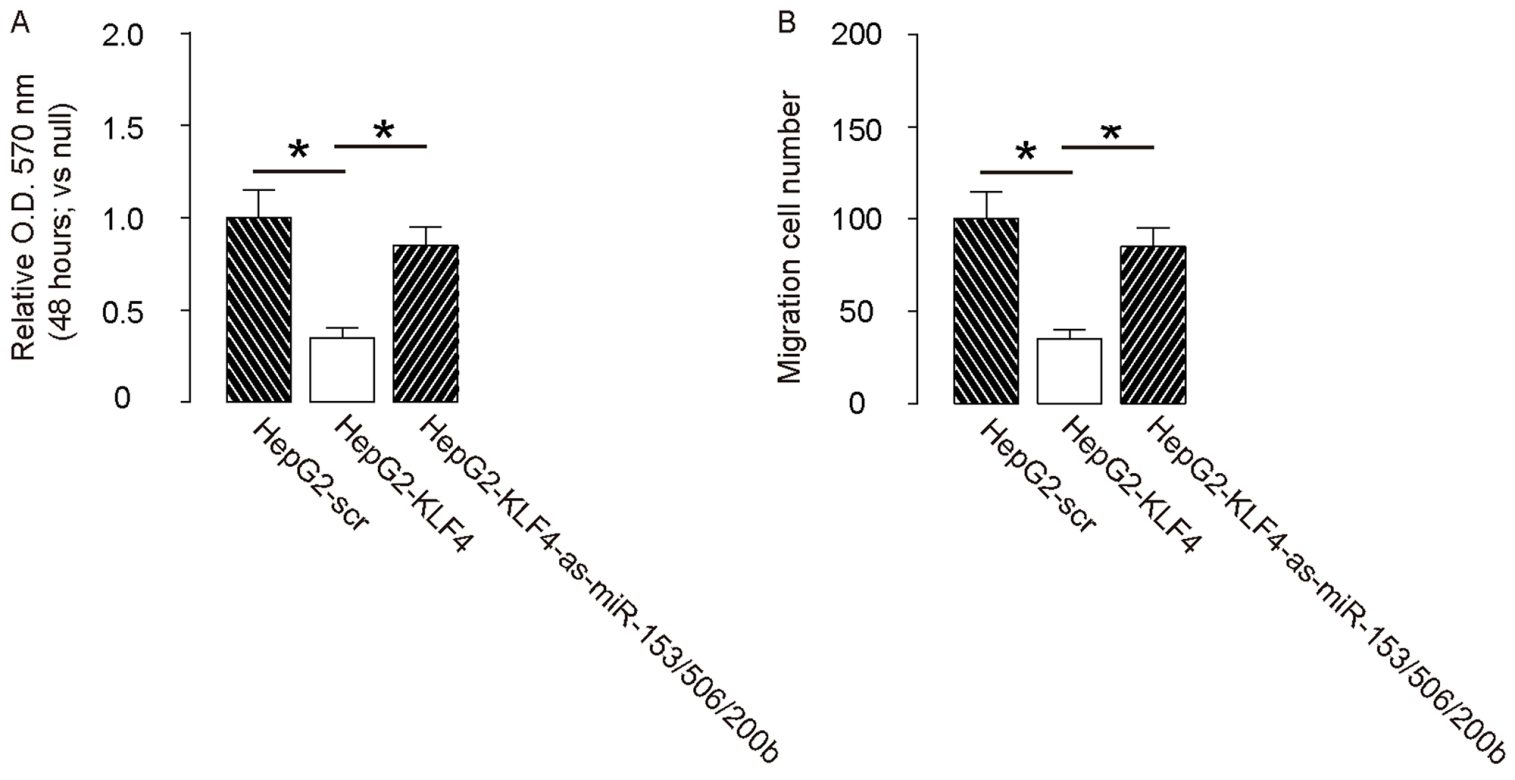
Depletion of miR-153, miR-506 and miR-200b abolishes the effects of KLF4 on HCC cell growth and invasion Depletion of miR-153, miR-506 and miR-200b abolished the effects of KLF4 on HCC cell growth in an MTT assay. **A.** and invasion in a transwell cell migration assay **B.** *p<0.05. N=5.

**Figure 7:**
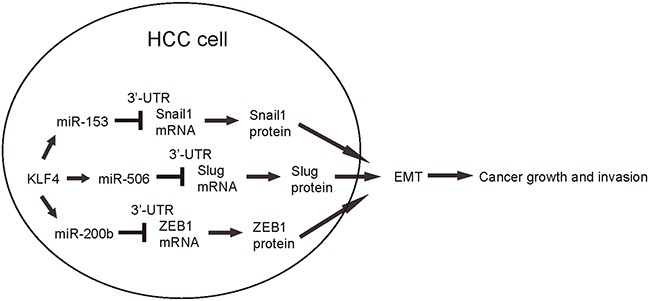
Schematic of the model KLF4 may suppress the HCC cell growth and invasion through miR-153, miR-506 and miR-200b-mediated suppression of Snail1, Slug and ZEB1, respectively.

In order to confirm that KLF4 may affect the HCC cell growth and invasion through miR-153, miR-506 and miR-200b, we overexpressed the antisense for miR-153, miR-506 and miR-200b in KLF4-expressing HepG2 cells (Figure 5A–5B), and compared to HepG2-KLF4 cells and HepG2-scr cells in both MTT and transwell cell migration assay. We found that miR-153, miR-506 and miR-200b depletion abolished the suppressive effects of KLF4 on HCC cell growth (Figure 6A) and invasion (Figure 6B), suggesting that KLF4 may inhibit the HCC cell growth and invasion through miR-153, miR-506 and miR-200b. Thus, this model is summarized in a schematic (Figure 7).

## DISCUSSION

Many miRNAs affect the growth and metastasis of HCC cells. Therefore, understanding of the aberrant expression of miRNAs in HCC cells may help to clarify the mechanisms underlying HCC metastasis and thus to improve therapy.

EMT enhances the carcinogenesis of HCC, for example, Sun et al. reported that stemness factor Sox2 correlates with metastasis and low survival rate in HCC, possibly through regulation of Slug transcription [29]. Previous studies have shown that EMT-associated proteins are regulated by miRNAs in HCC [30, 31]. For example, miR-200 family has been shown to inhibit ZEB1 and ZEB2 [19–23]. Moreover, Dhayat et al. reported that miR-200a and miR-200b were significantly downregulated in HCC, and could be served as an early marker for cirrhosis-associated HCC [32]. Wong et al. showed that miR-200b negatively regulated Rho/ROCK signaling pathway to suppress metastases of HCC [33]. Further, miR-506 has been shown to block Slug translation [24–27], and miR-153 has been shown to suppress Snail1 and ZEB2 [28]. A pioneer study has shown that expression of KLF4 in HCC cells sensitizes them to the anti-proliferative effects of VD3, possibly through regulation of EMT-associated events related to cell metastases and growth [18]. However, whether KLF4 may control EMT-associated proteins through miRNAs is unknown.

We have previously shown that the reduced levels of KLF4 in HCC correlate with increased levels of EMT-associated proteins, e.g. Snail1, Slug and ZEB1. The augment of these proteins is responsible for not only cancer invasion, but also allow the outgrowth of the tumor [18]. Moreover, in our previous work, we found that the suppression of KLF4 on EMT-associated factors Snail1, Slug and ZEB1 were more pronounced at protein levels, compared to mRNA levels, which suggests presence of post-transcriptional controls. Hence, in this study, we hypothesized that KLF4 may inhibit these EMT-associated proteins through modulation of miRNAs, which regulate gene expression at post-transcriptional level. Three miRNAs (miR-153, miR-506 and miR-200b) that target 3′-UTR of Snail1, Slug and ZEB1 mRNAs, respectively, were found to be induced by KLF4 overexpression, and suppressed by KLF4 depletion, in HCC cells. To further confirm that the bindings between these miRNAs and EMT-associated proteins are functional, we performed promoter luciferase assay. Moreover, expression of either miRNA significantly inhibited HCC cell growth and invasiveness, while the effect of combined expression of all 3 miRNAs was more pronounced. In a loss-of-function experiment, we suppressed the levels of miR-153, miR-506 and miR-200b in KLF4-overexpressing HCC cells, which completely abolished the effects of KLF4 on cell growth and invasion.

Since we have used 2 HCC cell lines and got essentially similar results independently, a possibility of these results to be cell-line-dependent is unlikely. Since our data were basically achieved from HCC cell lines, in future, examination of primary HCC specimen may be applied. Also, the analyses on the EMT-associated proteins were not complete, since some proteins like ZEB2, E-cadherin may be also examined in future studies. Further, the in vivo studies should be applied to confirm these in vitro findings.

Together, the findings in the current study highlight a suppressive role of KLF4 in regulation of EMT-enhanced tumor growth and invasion in HCC cells, and suggest that enhancement of either KLF4 or its downstream miRNAs may substantially improve the current treatment for HCC.

## MATERIALS AND METHODS

### Experimental protocol approval

All experimental protocols were approved by the Research Bureau of Department of Oncology, Shanghai First People's Hospital Affiliated to Shanghai Jiaotong University.

### HCC cell lines

HepG2 and Huh7 are two human HCC cell lines, which were purchased from American Type Culture Collection (ATCC, Rockville, MD, USA), and cultured in Dulbecco's modified Eagle's medium (DMEM, Invitrogen, Carlsbad, CA, USA) supplemented with 15% fetal bovine serum (FBS; Sigma-Aldrich, St Louis, MO, USA) in a humidified chamber with 5% CO_2_ at 37°C.

### Cell transfection

The coding sequence of human KLF4 was amplified using cDNA of human embryonic stem cells as a template, and cloned into pLVX-ZsGreen1-C1 vector (Clontech, Mountain View, CA, USA). The short hairpin small interfering RNA (shRNA) for KLF4 has been described before [18]. A scramble sequence was used as the mock control (scr). Human KLF4 target sequence: 5′-GCCAGAAAGCACTACAATC-3′; scr sequence: 5′-CTGCGATGCGCGTTCCGCTTA-3′. The sequences encoding miR-153, antisense (as)-miR-153, miR-506, as-miR-506, miR-200b, or as-miR-200b were similarly cloned into pLVX-ZsGreen1-C1 vector. The antisense sequences were single-stranded RNAs of complementary sequence to a specific miRNA. To control the infections with miRNAs, a null plasmid was prepared (null). In the current study, we did not detect the effects of scr or null on our results, compared to untreated cells. Thus, only data from scr- or null-treated cells were shown. Transfection with either KLF4, scr, shKLF4, miR-153, as-miR-153, null, miR-506, as-miR-506, miR-200b, or as-miR-200b-expressing plasmids was performed with Lipofectamine-2000 (Invitrogen). The success of transfection (nearly 100%) was assured by GFP expression of the transfected cells.

### MTT assay

For assay of cell viability, cells were seeded into 24 well-plate at 10000 cells per well and subjected to a Cell Proliferation Kit (MTT, Roche, Indianapolis, IN, USA), according to the instruction of the manufacturer. The MTT assay is a colorimetric assay for assessing viable cell number, taking advantage that NADPH-dependent cellular oxidoreductase enzymes in viable cells reduce the tetrazolium dye 3-(4,5-dimethylthiazol-2-yl)-2,5-diphenyltetrazolium bromide (MTT) to its insoluble formazan in purple readily being quantified by absorbance value (OD) at 570 nm. Experiments were performed 5 times.

### Transwell cell migration assay

Cells (10^4^) were plated into the top side of polycarbonate transwell filter coated with Matrigel in the upper chamber of the BioCoatTM Invasion Chambers (Becton-Dickinson Biosciences, Bedford, MA, USA) and incubated at 37°C for 24 hours. The cells inside the upper chamber with cotton swabs were then removed. Migratory and invasive cells on both the upper chamber and the lower membrane surface were fixed, stained with hematoxylin, and counted for 10 random 100X fields per well. Cell counts are expressed as the cell number in the lower membrane per total cell number (cells in both upper chamber and lower membrane). Five independent experiments were performed and the data are presented as mean ± standard deviation (SD).

### Western blot

Protein was extracted from the cultured cells with RIPA lysis buffer (1% NP40, 0.1% Sodium dodecyl sulfate (SDS), 100μg/ml phenylmethylsulfonyl fluoride, 0.5% sodium deoxycholate, in PBS) on ice. The supernatants were collected after centrifugation at 12000×g at 4°C for 20min. Protein concentration was determined using a BCA protein assay kit (Bio-rad, Shanghai, China), and whole lysates were mixed with 4×SDS loading buffer (125mmol/l Tris-HCl, 4% SDS, 20% glycerol, 100mmol/l Dithiothreitol (DTT), and 0.2% bromophenol blue) at a ratio of 1:3. Samples were heated at 100°C for 5 min and were separated on SDS-polyacrylamide gels. The separated proteins were then transferred to a PVDF membrane. The membrane blots were first probed with a primary antibody. After incubation with horseradish peroxidase-conjugated second antibody, autoradiograms were prepared using the enhanced chemiluminescent system to visualize the protein antigen. The signals were recorded using X-ray film. Primary antibodies were rabbit anti-KLF4, anti-Snail1, anti-Slug, anti-ZEB1 and anti-β-actin (Cell Signaling, San Jose, CA, USA). Secondary antibody was HRP-conjugated anti-rabbit (Jackson ImmunoResearch Labs, West Grove, PA, USA). β-actin was used as a protein loading control. The protein levels were first normalized to β-actin, and then normalized to the control.

### Quantitative real-time PCR (RT-qPCR)

Total RNA or miRNAs were extracted from cultured cells with miRNeasy mini kit or RNeasy kit (Qiagen, Hilden, Germany), respectively. Complementary DNA (cDNA) was randomly primed from 2μg of total RNA using the Omniscript reverse transcription kit (Qiagen). Quantitative real-time PCR (RT-qPCR) was subsequently performed in triplicate with a 1:4 dilution of cDNA using the Quantitect SyBr green PCR system (Qiagen) on a Rotorgene 6000 series PCR machine. All primers were purchased from Qiagen. Data were collected and analyzed using 2-ΔΔCt method for quantification of the relative mRNA expression levels. Values of genes were first normalized against β-actin, and then compared to the control.

### Luciferase-reporter activity assay

Luciferase-reporters were successfully constructed using molecular cloning technology. Target sequence was inserted into pGL3-Basic vector (Promega, Madison, WI, USA) to obtain pGL3-Snail1-3′UTR, pGL3-Slug-3′UTR or pGL3-ZEB1-3′UTR, which contain the miR-153, miR-506 or miR-200b binding sequence, respectively. MiRNA-modified HCC cells were seeded in 24-well plates for 24 hours, after which they were transfected with 1μg of Luciferase-reporter plasmids per well using PEI Transfection Reagent. Then luciferase activities were measured using the dual-luciferase reporter gene assay kit (Promega), according to the manufacturer's instructions.

### Statistical analysis

All statistical analyses were carried out using the SPSS 18.0 statistical software package. All data were statistically analyzed using one-way ANOVA with a Bonferroni correction, followed by Fisher's exact test. All values are depicted as mean ± standard deviation from 5 individuals and are considered significant if p < 0.05.
